# Direct health implications of e-cigarette use: a systematic scoping review with evidence assessment

**DOI:** 10.3389/fpubh.2024.1427752

**Published:** 2024-07-29

**Authors:** Juan S. Izquierdo-Condoy, Patricio Naranjo-Lara, Estefanía Morales-Lapo, Marlon R. Hidalgo, Andrea Tello-De-la-Torre, Eduardo Vásconez-Gonzáles, Camila Salazar-Santoliva, Valentina Loaiza-Guevara, Wendy Rincón Hernández, Diego Alexander Becerra, María Belén Delgado González, Andrés López-Cortés, Esteban Ortiz-Prado

**Affiliations:** ^1^One Health Research Group, Faculty of Medicine, Universidad de las Américas, Quito, Ecuador; ^2^Facultad de Medicina, Fundación Universitaria Autónoma de las Américas, Pereira, Colombia; ^3^Fundacion Universitaria San Martín, Bogota, Colombia; ^4^Facultad de Ciencias de la Salud, Universidad del Quindío, Armenia, Colombia; ^5^Cancer Research Group (CRG), Faculty of Medicine, Universidad de las Americas, Quito, Ecuador

**Keywords:** E-cigarettes, health effects, tobacco harm reduction, chronic effects of vaping, systematic review

## Abstract

**Background:**

E-cigarettes are often marketed as a less harmful alternative to traditional tobacco cigarettes. Despite their popularity, the evidence regarding their effects on human health remains unclear and is filled with complexities.

**Objectives:**

This systematic review aims to elucidate the direct effects of electronic cigarette use on human health, carefully distinguishing between the specific characteristics of the populations studied.

**Methodology:**

Adhering to the PRISMA guidelines, we conducted a comprehensive search in PubMed/Medline, Web of Science, Scopus, and Google Scholar databases without date restrictions, including articles in both Spanish and English. This approach enabled the identification and analysis of primary studies to understand the direct effect of electronic cigarettes on human health.

**Results:**

A total of 33 studies were included that evaluated cardiovascular, pulmonary, renal, weight and fertility effects. Only five studies analyzed e-cigarettes in healthy populations and seven studies compared healthy individuals against smokers. The effects evaluated on smokers or former tobacco smokers were apparently positive, however, among healthy individuals, increased heart rate, mean arterial pressure, oxidative stress, alteration of respiratory epithelial cells and increased airflow resistance were found.

**Conclusion:**

Smokers or former smokers who switch to e-cigarettes may reduce their exposure to carcinogens and lower their risk of developing severe health issues associated with conventional smoking. However, in healthy individuals who have never smoked traditional cigarettes, the use of e-cigarettes introduces several cardiovascular and respiratory adverse effects. These findings suggest that while e-cigarettes can be a strategic harm reduction tool for smokers, they are not a safe option for non-smokers.

## 1 Introduction

Smoking has been associated with several negative health effects since the early 1930s (1). It was not until the 1950s that Dr. Richard Doll and Dr. A. Bradford Hill published a series of influential studies highlighting the negative health effects of tobacco, including the renown British Doctors Study (2). Since then, public health advocates have actively sought methods to reduce tobacco consumption and its associated risks. This effort has been supported by numerous initiatives from the anti-tobacco industry aimed at decreasing smoking rates. Strategies have included the implementation of taxation, prohibition of smoking in public areas, strict regulatory controls, and the promotion of pharmacological nicotine replacement therapies (3, 4). In 2004, electronic cigarettes (EC) were introduced to the market as a healthier alternative for chronic smokers dependent on nicotine, allowing them to smoke without the risks of tar and other toxic tobacco compounds (5). Due to their ability to generate vapor instead of smoke, electronic cigarettes (ECs) have gained popularity among those seeking to quit traditional smoking (6). They also appeal to adolescents and young adults, attracted by marketing campaigns that tout ECs as a safer alternative to traditional cigarettes and an effective tool for cessation (7–10). This perceived safety stems from the fact that traditional cigarettes require the combustion of paper and tobacco to generate smoke, which carries tar, nicotine, carbon monoxide, and other harmful substances into the lungs. In contrast, ECs do not involve combustion (8, 11).

The global e-cigarette market is projected to grow from $22.5 billion in 2022 to $47.5 billion in 2028, with a compound annual growth rate (CAGR) of 13.5% from 2023 to 2028 (12). Prevalence among adults is approximately 10%, while it reaches 11.8% among middle and high school students. Notably, usage rates in these student groups surged by 10.5 and 27.5%, respectively, in 2019 (13, 14). In 2020, it was estimated that 68 million adults used electronic cigarettes, predominantly those aged 18–24 years (15, 16).

Although vaping electronic cigarettes is generally considered safer than smoking traditional tobacco, there is significant concern regarding the variety of compounds that can be combined in these devices. Most liquids used in e-cigarettes contain propylene glycol and glycerol, which are irritants to the respiratory tract (17). Furthermore, the degradation of e-cigarette vapor can produce formaldehyde, a carcinogenic substance. Studies have also shown an increase in inflammatory markers in the respiratory tract up to 10 times greater than those found in traditional cigarette smokers (18, 19). Some e-cigarette cartridges also contain flavor enhancers like diacetyl, which, when inhaled, not only increases the risk of addiction but can also cause tissue damage such as bronchiolitis obliterans, a serious respiratory condition often referred to as “popcorn lung” (7). Despite some ECs containing no nicotine, the most popular ones feature nicotine levels ranging from 6 to 24 mg/mL, and even up to 100 mg/mL, making them highly addictive and resulting in higher blood nicotine levels compared to traditional smoking (7, 11). These high levels of nicotine not only raise concerns about addiction but may also contribute to the numerous health issues associated with EC use.

The safety of e-cigarette consumption is not well-established, and evidence shows that ECs can cause lung inflammation and damage, disrupt lung epithelial cell function, and irritate the eyes, nose, and throat. Additionally, aerosolized nicotine from vaping has been linked to increased thromboembolic activity and impaired dilation and relaxation of small blood vessels (7, 11).

Given the ongoing debates about the health implications of electronic cigarette use, this systematic review aims to thoroughly investigate the direct effects of electronic cigarette use on human health, considering the characteristics of the populations studied and the duration of exposure.

## 2 Materials and methods

### 2.1 Study design

We conducted a systematic review comprising primary source studies, including clinical trials, cross-sectional studies, cohorts, case controls, case series, and clinical case reports. Secondary source studies such as systematic reviews, meta-analyses, literature reviews, and narrative reviews were excluded, as were letters to the editor, comments, special articles, and editorials. We followed the Preferred Reporting Items for Systematic Reviews and Meta-Analyses (PRISMA) methodology, a recommended guide for conducting systematic reviews and meta-analyses. This review did not have a registered protocol in the International prospective register of systematic reviews (PROSPERO), which does not accept registrations for scoping reviews, literature reviews, or mapping reviews.

### 2.2 Search strategies

We conducted an in-depth bibliographic search in English to encompass the broadest scope of academic literature. We utilized several key databases and libraries, including PubMed/Medline, Web of Science, Scopus, and Google Scholar. Additionally, we employed a snowball strategy to review the reference lists of relevant articles for any overlooked studies. Our literature search targeted primary studies published before June 2023. To execute the search, we used the following index terms, keywords, and Boolean operators: (“Electronic Nicotine Delivery Systems” OR “E-Cigarettes” OR “Electronic Cigarettes” OR “Vaping” OR “Nicotine Vaping” OR “Vape”) AND (“Health effects” OR “Toxicity” OR “Health Risk” OR “Physiology”) AND (“Effects, Acute” OR “Effects, Long-Term”) in the title (TI) or abstract (AB).

### 2.3 Selection criteria

#### 2.3.1 Inclusion criteria

Primary studies examining the direct short-term or long-term health effects of using or consuming e-cigarettes, including impacts on the cardiovascular, respiratory, and other systems.

Studies conducted on human subjects.

#### 2.3.2 Exclusion criteria

Studies examining the health effects of e-cigarette use conducted on animals.

Research focusing on the indirect health effects of e-cigarette use, such as assistance in quitting traditional tobacco cigarettes.

Studies that investigate the effects of consuming substances other than e-cigarettes, such as drugs or tobacco.

Secondary research or reviews on the health impacts of e-cigarette use.

**Figure 1:**
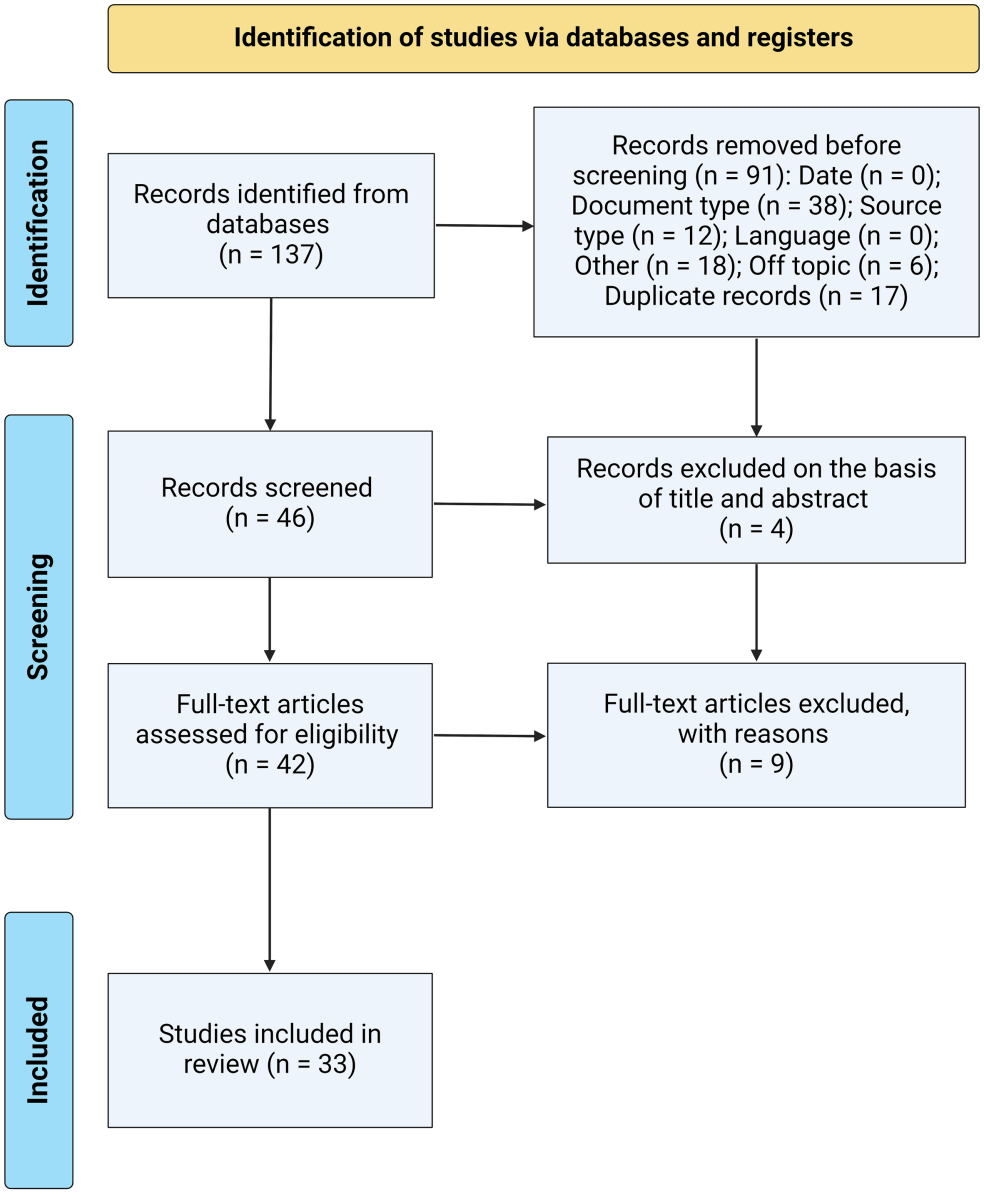
PRISMA flowchart illustrating the study selection process for this systematic review, detailing the number of studies screened, assessed for eligibility, and included in the review, with reasons for exclusions at each stage.

The bibliographic search initially yielded a total of 137 papers. In the first screening phase, 74 studies were eliminated, primarily due to the type of document (*n* = 38), and 17 studies were eliminated due to duplicates. Of the 46 remaining papers, four studies were excluded due to limitations found in the title or abstract. Finally, the 42 eligible papers were reviewed in their entirety, and 33 studies were included in this investigation. Figure 1 shows the selection process based on the PRISMA flow chart of the studies analyzed in this manuscript.

### 2.4 Bias assessment

To minimize the risk of bias, four members of the research team (JSIC, MH, EML, and PNL) independently performed the data extraction process at different times. Any discrepancies encountered during the data collection phase were resolved through discussion until consensus was reached among all team members. This method was implemented to ensure the accuracy and reliability of our findings.

### 2.5 Data synthesis

A comprehensive review was conducted on all manuscripts that met the established selection criteria. For cohort studies, we utilized the Newcastle-Ottawa Quality Assessment Scale for quantitative analysis, the Newcastle-Ottawa Scale was developed to assess the quality of nonrandomized studies with its design, content and ease of use directed to the task of incorporating the quality assessments in the interpretation of meta-analytic results (20). For cross-sectional studies, the Joanna Briggs Institute (JBI) critical appraisal checklist for analytical cross-sectional studies was employed, the JBI’s critical appraisal tools assist in assessing the trustworthiness, relevance and results of published papers (21). Additionally, for Randomized Controlled Trials (RCTs), we applied the JBI critical appraisal checklist specific to RCTs (21) (see Supplementary material 1).

The information from these manuscripts was meticulously organized and synthesized into descriptive tables. This format was chosen to present our findings clearly and concisely, facilitating ease of understanding for the reader.

## 3 Results

### 3.1 Literature review and quality assessment

A total of 33 studies were included in this systematic review, of which 15 were cohort studies. Among these, 11 were of good quality and four were of acceptable quality. In addition, 17 studies were randomized controlled trials, with 10 of high quality and seven of moderate quality. Finally, only one cross-sectional study was identified, and it was classified as high quality.

### 3.2 Cardiovascular effects

#### 3.2.1 Effects on hemodynamics

E-cigarette use has been linked to increased heart rate and blood pressure. This was observed in a combined analysis of nine randomized studies (22–30) and one prospective study (31) which investigated the short-term effects of e-cigarettes on healthy subjects, both with and without a history of tobacco use. Significant increases in heart rate, systolic and diastolic blood pressure, and mean arterial pressure were noted following acute inhalation of e-cigarettes, regardless of nicotine content. Conversely, a randomized study by D’Ruiz et al. involving 105 subjects who switched either completely or partially from tobacco cigarettes to electronic cigarettes demonstrated that electronic cigarette use over 5 days led to reduced blood pressure and heart rate in most participants (32).

Conversely, among users who transitioned from tobacco cigarettes to e-cigarettes, evidence from three studies, including a prospective randomized controlled trial involving active tobacco smokers, indicates a significant decrease in systolic blood pressure and resting heart rate after 1 month of e-cigarette use, with or without nicotine. This effect was particularly noted in participants who had smoked more than 20 pack-year of tobacco (33). Similarly, a randomized controlled clinical trial involving 263 tobacco smokers who switched to e-cigarettes demonstrated a statistically significant decrease in blood pressure and heart rate at the 1-month follow-up. However, these results did not maintain statistical significance at the 3-month follow-up (34).

Regarding long-term exploration through *post hoc* analysis of the ECLAT study, a 12-month prospective, randomized, controlled, double-blind trial in smokers with no intention to quit tobacco smoking who switched to e-cigarettes with and without nicotine, it was found that those who switched to e-cigarettes experienced a statistically significant reduction in long-term systolic blood pressure, with this reduction being more pronounced in smokers with elevated baseline blood pressure (35). Regarding the long-term effects of e-cigarette smoking on cardiac autonomic tone, a cross-sectional study comparing cases (e-cigarette users) and controls (healthy individuals with no history of smoking) who had used nicotine-containing e-cigarettes on most days for at least 1 year, showed a significant increase in sympathetic activity compared to the control group (30). Additionally, an evaluation of effects in tobacco smokers found an improvement in baseline acetylcholine and mean arterial pressure 3 and 6 months after starting e-cigarette replacement (*p* < 0.05) (36) (Table 1).

**Table 1:** Systematic summary of evidence of studies assessing the cardiovascular effects of e-cigarette use.

Author	Study design	Population	Evaluated parameter	EC effect
Effects on hemodynamics				
Gonzalez and Cooke (22)	Cohort	15 healthy subjects	^a^ Mean arterial pressure, heart rate, and sympathetic activity.	Increased ^c^: mean arterial pressure and heart rate. Decreased muscle sympathetic nerve activity.
Biondi-Zoccai et al. (23)	Randomized trial	20 smokers	^a^ Blood pressure.	Increased ^c^: systolic, diastolic, and mean blood pressure.
Antoniewicz et al. (24)	Cohort	17 healthy subjects	^a^ Blood pressure, heart rate.	Increased ^d^: systolic, and diastolic blood pressure.
				Increased ^c^: heart rate.
Yan and D’Ruiz (25)	Randomized Trial	23 smokers	^a^ Heart rate, systolic and diastolic blood pressure.	Increased ^c^: systolic and systolic blood pressure, heart rate.
Dimitriadis et al. (26)	Randomized controlled trial	12 healthy smokers	^a^ Mean arterial pressure and heart rate.	Increased ^c^: mean arterial pressure and heart rate.
Cooke et al. (27)	Randomized trial	20 healthy subjects	^a^ Blood pressure.	Increased ^c^: systolic and diastolic pressure in sitting and head-up position.
Spindle et al. (28)	Cohort	30 healthy smokers	^a^ Hearth rate.	Increased ^c^: heart rate.
Chaumont et al. (29)	A randomized crossover trial	25 smokers	^a^ Systolic and diastolic blood pressures, and heart rate.	Increased ^c^: systolic and diastolic blood pressures, and heart rate
Moheimani et al. (30)	Randomized trial	33 healthy subjects	^b^ Heart rate.	Increased ^c^: heart rate.
Kerr et al. (31)	Cross-over study	20 smokers	^a^ Heart rate.	Increased ^c^: heart rate.
D’Ruiz et al. (32)	Randomized trial	105 smokers	^a^ Systolic and diastolic blood pressure, heart rate.	Decrease ^c^: Systolic and diastolic blood pressure, heart rate.
George et al. (33)	Cohort	114 healthy smokers	^b^ Heart rate and blood pressure.	Decrease ^e^: systolic blood pressure.
Veldheer et al. (34)	Randomized controlled trial	263 smokers	^b^ Blood pressure and heart rate.	Decrease ^c^: Systolic blood pressure, and heart rate.
				
Farsalinos et al. (35)	Cohort	300 smokers	^b^ Blood pressure and heart rate.	Decrease ^d^: Systolic blood pressure.
Klonizakis et al. (36)	Randomized controlled trial	248 smokers	^b^ Mean arterial pressure.	Decrease ^d^: Mean arterial pressure.
Polosa et al. (37)	Cohort	Nine smokers	^b^ Systolic and diastolic blood pressure, heart rate.	No changes were found in systolic and diastolic blood pressure, and heart rate.
		12 never smokers		
Impact on arterial vasculature				
Antoniewicz et al. (24)	Cohort	17 healthy subjects	^a^ Arterial stiffness (by pulse wave velocity and AIx75).	Increased ^c^: pulse wave velocity and AIx75.
George et al. (33)	Cohort	114 healthy smokers	^b^ Arterial stiffness (by pulse wave velocity).	Decrease ^d^: pulse wave velocity.
Klonizakis et al. (36)	Randomized controlled trial	248 smokers	^b^ Microvascular assessment (by acetylcholine, and sodium nitroprusside).	Increased ^d^: Acetylcholine, and sodium nitroprusside.
Influence on cardiovascular biomarkers				
Flouris et al. (38)	Randomized Trial	30 (15 smokers, 15 never-smokers)	^a^ Complete blood count.	Increase ^c^: white blood cell, lymphocyte, and granulocyte counts.
Biondi-Zoccai et al. (23)	Randomized Trial	20 smokers	^a^ Oxidative stress, antioxidant reserve, platelet function.	Increase ^c^: soluble Nox2-derived peptide, 8-iso-prostaglandin F2α-III, H2O2, ligand CD40, P-selectin
				Decreased ^c^: vitamin E
Mobarrez et al. (39)	Cohort	17 healthy “occasional” smokers	^a^ Platelet and endothelial derived extracellular vesicles.	Increase ^c^: CD40-ligand, soluble P-selectin and platelet derived extracellular vesicles.
Nocella et al. (40)	Cohort	40 (20 smokers – 20 nonsmokers)	^a^ Platelet function.	Increase ^c^: soluble CD40-ligand, soluble P-selectin, platelet aggregation.
Chaumont et al. (29)	A Randomized Crossover Trial	25 smokers	^a^ Oxidative stress.	Increased ^c^: plasma myeloperoxidase.
Kerr et al. (31)	Cross-over study	20 smokers	^a^ Blood circulating microparticles (particularly platelet microparticles).	Increase ^c^: Platelet microparticles.
Antoniewicz et al. (41)	Randomized Trial	16 smokers	^a^ Blood level of endothelial progenitor cells.	Increase ^c^: levels of endothelial progenitor cells.
Moheimani et al. (30)	Randomized Trial	33 healthy subjects	^b^ Oxidative stress.	No differences in changes in any measures of oxidative stress (low-density lipoprotein oxidizability: paraoxonase-1: igh-density lipoprotein antioxidant index).
Effects on cardiac function				
Farsalinos et al. (42)	Randomized Trial	36 healthy “heavy” smokers	^a^ Left ventricular function (by echocardiographic).	Decrease ^c^: isovolumetric relaxation time corrected-to-heart rate, myocardial performance index, and myocardial performance index and tissue doppler.
		40 electronic cigarette users		
Alzahrani et al. (43)	Cross-sectional study	28,128 smokers – 41,239 nonsmokers	(Unknown) Myocardial infarction.	Daily e-cigarette use was independently associated with increased odds of having had a myocardial infarction
Vansickel et al. (44)	Randomized controlled trial	32 smokers	^a^ Plasmatic nicotine and CO.	Not changes were found in NPRO and Hydro EC in plasma nicotine and plasma CO.

^a^Acute effects of electronic cigarettes; ^b^Long-term effects of electronic cigarettes; ^c^NICOTINE attributed effects; ^d^Effects found with and without nicotine exposure; ^e^Effects found without nicotine exposure. All effects described in the table as “increase” or “decrease” correspond to statistically significant results found in the included studies. Some of the studies included in the table may have evaluated several parameters, in which case the same study may be mentioned more than once in the table.

#### 3.2.2 Impact on arterial vasculature

Regarding the effects on vasculature, three studies were included. A randomized crossover study of occasional tobacco cigarette users demonstrated an acute increase in vascular stiffness after inhaling e-cigarettes with and without nicotine, as evidenced by increased AI75 (pulse wave augmentation index at 75%) and PWV (pulse wave velocity) (24). However, George et al. reported that in tobacco smokers, arterial stiffness (PWV-dependent) improved after 1 month of switching from tobacco cigarettes to e-cigarettes, with or without nicotine. They also noted a significant enhancement in endothelial function as measured by flow-mediated dilatation (FMD) (33). In contrast, Biondi et al. observed a significant deterioration in FMD immediately after using nicotine-containing e-cigarettes (23).

Furthermore, long-term e-cigarette use in smokers attempting to quit has been associated with improved flow-mediated dilation at 3 and 6 months (*p* < 0.001) (36) (Table 1).

#### 3.2.3 Influence on cardiovascular biomarkers

Acute effects on biomarkers across five studies involving healthy populations with a history of smoking, including a randomized crossover study between tobacco smokers and non-smokers, demonstrated that active e-cigarette use in smokers and passive use in healthy individuals did not affect blood count markers. However, both active and passive tobacco users exhibited an increase in white blood cell, lymphocyte, and granulocyte counts for at least 1 h (38). Additionally, Biondi et al. revealed that both tobacco and electronic cigarette inhalation significantly increased markers of oxidative stress (sNox2-dp, H2O2, and 8-iso-PGF2a levels) and platelet activity (sCD40L and soluble P-selectin), and decreased markers of NO bioavailability and antioxidants (Vitamin E) in tobacco smokers classified as healthy (23), Similarly, Mobarrez et al. observed an increase in endothelial and platelet-derived vesicles (expressing increased P-selectin) 4 h after the consumption of nicotine-containing electronic cigarettes, and noted an increase in CD40 ligand even in nicotine-free e-cigarettes (39). Furthermore, Nocella et al. comparing the acute impact of tobacco versus e-cigarettes with equivalent nicotine content, found that within 5 min of smoking or vaping, both tobacco smokers and healthy individuals exhibited a statistically significant increase in sCD40L, sP-selectin, and platelet aggregation levels in a single-blind crossover trial (40). Conversely, a randomized single-blind, three-period measurement in smokers showed that exposure to nicotine-free electronic cigarettes did not lead to acute changes in cardiovascular oxidative stress parameters; however, exposure with nicotine resulted in increased arterial stiffness and plasma myeloperoxidase (*p* < 0.05) (29). Additionally, a prospective cross-sectional study reported an increase in platelet microparticles (PMPs) (*p* < 0.001) following electronic cigarette consumption (31).

In a randomized crossover trial comparing healthy subjects who smoked tobacco (maximum of 10 cigarettes per month), the acute use of nicotine-containing e-cigarettes significantly increased levels of circulating endothelial progenitor cells (flow cytometry) to the same magnitude as after smoking a traditional cigarette (41). This group also showed an increase in endothelial vesicles of platelet and endothelial origin 4 h after exposure to nicotine-containing e-cigarettes (39).

Regarding long-term effects, Moheimani et al. found that low-density lipoprotein (LDL) oxidizability was higher in e-cigarette users compared to the control group (healthy) (*p* = 0.78), while paraoxonase-1 activity, which protects against oxidative stress, tended to be lower in e-cigarette users (*p* = 0.72). Furthermore, markers of inflammation such as high-density lipoprotein antioxidant index, fibrinogen, and C-reactive protein did not differ between groups (30) (Table 1).

#### 3.2.4 Effects on cardiac function

The immediate cardiac effects of e-cigarettes compared to traditional tobacco were assessed through echocardiography. The results indicated that the e-cigarette group exhibited a lower heart rate-corrected isovolumetric relaxation time (IVRTc) (*p* = 0.011) and tissue Doppler flow (MPIt) (*p* = 0.019). These findings suggest a lack of immediate relaxing effects on the left ventricular musculature typically observed in smokers (42). Furthermore, an analysis of cross-sectional data from the US National Health Interview Surveys (NHIS) explored the relationship between e-cigarette use and myocardial infarction. This study found that daily e-cigarette use was independently associated with increased odds of having suffered a myocardial infarction (43) (Table 1).

### 3.3 Impact on respiratory health

#### 3.3.1 Changes in respiratory epithelial cells

Two randomized trials have evaluated the effects of e-cigarettes on respiratory epithelial cells. In a randomized pilot trial, Song et al. studied healthy non-smoking individuals exposed to nicotine-free, unflavored e-cigarettes containing 50% propylene glycol and 50% vegetable glycerin. They found that propylene glycol caused low-grade lung inflammation after 1 month of exposure, but no changes in gene expression in lung cells were observed (45). Conversely, Staudt et al., in a trial with healthy non-smoking subjects exposed to both nicotine-containing and nicotine-free e-cigarettes, noted genetic changes in small airway epithelial cells, alveolar macrophages, and lower airway mononuclear phagocytes after short exposure to EC aerosols. This study also reported alterations in alveolar macrophages, including changes in the systemic inflammatory response, impaired phagocytic capacities, and increased susceptibility to bacteria, particularly *S. pneumoniae* (46) (Table 2).

**Table 2:** Systematic summary of evidence of studies assessing the respiratory effects of e-cigarette use.

Publication	Study design	Population	Evaluated parameter	EC effects
Changes in respiratory epithelial cells				
Song et al. (45)	Clinical trial	30 never smokers	^b^ Inflammation in the human lung (bronchoalveolar lavage cells and cytokines).	No significant differences in changes of bronchoalveolar lavage inflammatory cell counts or cytokines.
Staudt et al. (46)	Cohort	10 never smokers	^a^ Lung function (small airway epithelium and alveolar macrophages)	Altered transcriptomes of small airway epithelium cells and of the alveolar macrophages^d^.
Effects on lung function				
				
Ferrari et al. (47)	Randomized trial	10 smokers – 10 nonsmokers	^a^ Pulmonary function (FEV1, FVC, FEV1/FVC, and PEF).	Decrease ^e^: FEF-25 in smokers.
Chaumont et al. (48)	Randomized trial	30 excusive e-cigarette users	^a^ Lung function (by FEV, FEF-75%, FEF-50%, DLCO and DLNO).	No changes were found in FEV, FEF-75%, FEF-50%, DLCO, DLNO, and DLNO/DLCO.
Flouris et al. (49)	Randomized trial	15 smokers – 15 nonsmokers	^a^ Lung function (by FEV1/FVC).	Decrease ^c^: 2.3% of FEV1/FVC.
Dicpinigaitis et al. (50)	Cohort	30 smokers	^a^ Cough reflex sensitivity (by capsaicin cough challenge).	Increase ^c^: concentration of capsaicin inducing five or more coughs (C5).
Cioe et al. (51)	Cohort	19 smokers + HIV-positive	^b^ Cardiopulmonary function, respiratory symptoms, and carbon monoxide.	Decrease ^c^: cough, wheezing, shortness of breath, mean CO.
D’Ruiz et al. (32)	Cohort	105 smokers	^a^ Pulmonary function (FVC, FEV1).	No changes were found in FVC, FEV1.
Antoniewicz et al. (24)	Cohort	17 healthy subjects	^a^ Flow resistance (impulse oscillometry).	Increased ^c^: flow resistance.
Kerr et al. (31)	Cohort	20 smokers	^a^ Respiratory function (standard spirometer).	Decreased ^c^: peak expiratory flow and exhaled carbon monoxide levels.
Veldheer et al. (34)	Randomized trial	263 smokers	^b^ Lung function.	No differences were found in FEV1, FVC, FEV1/FVC ratio, and FEF25–75.
Polosa et al. (37)	Cohort	9 smokers – 12 never smokers	^b^ Lung function.	No changes were found FEV1, FVC, FEV1/FVC ratio, and FEF25–75^d^.
Effects on respiratory biomarkers				
D’Ruiz et al. (32)	Cohort	105 smokers	^a^ Exhaled CO and NO.	Decrease ^c^: exhaled CO and NO levels.
				
O’Connell et al. (52)	Randomized Trial	105 healthy smokers	^a^ Exhaled CO and NO.	Decrease ^c^: exhaled CO.
				Increase ^c^: exhaled NO.
Campagna et al. (53)	Cohort	300 smokers	^b^ Fractional nitric oxide concentration in exhaled breath (FeNO), exhaled carbon monoxide (eCO).	Increase (with or without nicotine): FeNO.
				Decrease ^d^: eCO.
Polosa et al. (37)	Cohort	9 smokers – 12 never smokers	^b^ Exhaled CO and FeNO.	No changes were found in eCO and FeNO^d^.
Veldheer et al. (34)	Randomized trial	263 smokers	^b^ Exhaled CO.	No differences were found in exhaled CO.

^a^Acute effects of electronic cigarettes; ^b^Long-term effects of electronic cigarettes; ^c^nicotine attributed effects; ^d^Effects found with and without nicotine exposure; ^e^Effects found without nicotine exposure. All effects described in the table as “increase” or “decrease” correspond to statistically significant results found in the included studies. Some of the studies included in the table may have evaluated several parameters, in which case the same study may be mentioned more than once in the table.

#### 3.3.2 Effects on lung function

Among the acute effects on lung function, five studies evaluated them from different perspectives. Ferrari et al. observed that non-smokers using nicotine-free electronic cigarettes (EC) had a lower exhaled fraction of carbon monoxide (FeCO) compared to smokers; however, the exhaled fraction of nitric oxide (FeNO) showed no significant difference. Significant increases in both FVC (0.5–3.1%) and FEV1 (1.5–6%) were observed for both groups (tobacco and electronic cigarette users) over 5 days under various conditions. Furthermore, among non-tobacco smokers, exposure to electronic cigarettes resulted in a decrease in forced expiratory flow at 75% (FEF75), indicating that nicotine-free ECs do not produce significant short-term changes in lung functions in non-smokers but have small effects on different lung functions in tobacco smokers (47). Two randomized trials examined individuals who switched from tobacco cigarettes to electronic cigarettes, revealing that using ECs for 5 days did not lead to negative respiratory health outcomes, with spirometry findings showing no significant effect on airflow obstruction or lung function after EC use (32). Chaumont et al. reported that cessation of e-cigarette use appeared to improve the lung inflammation profile, though it did not affect spirometry variables (FEV1), lung membrane diffusing capacity, or lung capillary volume; additionally, nicotine-containing e-cigarettes did not decrease forced expiratory flow (FEF) compared with cessation of e-cigarette use (48). Flouris et al., in a study on the short-term impact of active and passive use of nicotine-containing ECs versus conventional tobacco, found similar cotinine levels in users as in conventional tobacco smokers, with most changes occurring in smokers. They noted small changes in pulmonary function (increase in FEV_1_/FVC, FVC, PEF, FEF_25_-_75_), an increase in exhaled carbon monoxide (CO), and serum nicotine, and a decrease in exhaled fraction of nitric oxide (FeNO), although these values were lower than those found in regular tobacco users (49).

Regarding long-term effects in the general population with a history of smoking, a controlled 1-year follow-up study found that changes in exhaled nitric oxide (FeNO) were significantly correlated with those of exhaled carbon monoxide (COe) throughout the follow-up, mainly a relative increase in the first 3 months followed by a significant increase at 6 and 12 months (53).

Regarding short-term respiratory symptoms, Dicpinigaitis et al. found a significant inhibition of cough reflex sensitivity as measured by the cough provocation test (capsaicin inhalation) in 30 individuals with no history of smoking after a single exposure to e-cigarette vapor; this effect was transient, as cough reflex sensitivity returned 24 h after EC use (50). They also examined 20 HIV-positive smokers who completely switched from tobacco cigarettes to EC during an 8-week follow-up, demonstrating clinical improvement in symptoms such as coughing, wheezing, and shortness of breath, a decrease in daily cigarette consumption, and complete cessation in seven participants (51). Campagna et al. noted that, in the long term (1 year), respiratory symptoms rapidly disappeared with the use of electronic cigarettes, both in individuals who quit smoking completely and those who reduced tobacco consumption (53) (Table 2).

#### 3.3.3 Effects on respiratory biomarkers

Regarding the effects on respiratory biomarkers, two studies explored this influence in the short term. A randomized trial of dual use of tobacco and e-cigarettes (with different flavors) showed a decrease in harmful or potentially harmful biomarkers for polycyclic aromatic hydrocarbons (PAHs such as pyrene) in users of electronic cigarettes, which were reduced by 62–69%. The levels of Tobacco Specific Nitrosamines (TSNAs), such as 4-(methylnitrosamino)-1-(3-pyridyl)-1-butanol (NNAL) and N-nitrosinornicotine (NNN), were reduced by 62–64 and 87–93%, respectively, as well as a reduction in the levels of volatile organic compounds. As for CO and NO measurements, the decrease in the e-cigarette cessation group saw a reduction of 89%. In addition, exhaled nitric oxide measurements evidenced an increase from day 1 to 5 mainly in the EC use and tobacco cessation groups (52). In contrast, Veldheer et al. evaluated the influence of electronic vs. non-electronic cigarette use (non-aerosol producing so-called cigarette substitutes) in tobacco smoking subjects, finding no relevant changes in terms of lung function after 3 months of e-cigarette use. In addition, it was determined that EC consumption reduced the number of daily cigarettes consumed and cigarette dependence in smoking subjects (34) (Table 2).

### 3.4 Effects on other tissues and organs

#### 3.4.1 Renal effects

Two randomized trials evaluated the short-term effects of e-cigarette use at the urinary level in former tobacco smokers and current nicotine e-cigarette users (at least 1 year). The effects of e-cigarette cessation with and without nicotine at the urinary level showed a decrease in the excretion of propylene glycol, 3-hydroxyisovalerate, and pyruvate, for those who performed the complete cessation sessions of vaping. While within the nicotine vaping sessions, the urinary excretion of trimethylamine oxide and hippurate were lower. Finally, the excretion of N-phenylacetyl glycine was lower in nicotine sessions compared to complete cessation of vaping (48). Similarly, among current tobacco smokers, e-cigarette smokers, and controls, it was evidenced that urinary excretion of biomarkers of exposure in individuals who ceased regular and electronic cigarette smoking decreased by 66–98%, including NNAL (4-(methylnitrosamino)-1-(3-pyridyl)-1-butanol), carboxyhemoglobin, nicotine, and its metabolites (52) (Table 3).

**Table 3:** Systematic summary of evidence on the effects of e-cigarettes on various tissues and organs.

Publication	Study design	Population	Evaluated parameter	EC effects
Renal effects				
Chaumont et al. (48)	Randomized trial	30 excusive e-cigarette users	^a^ Urine metabolite.	Increase ^c^: 3-hydroxyisovalerate, urine pyruvate. Decrease ^c^: trimethylamine oxide, urine Hippurate, and Phenylacetyl-glycine.
O’Connell et al. (52)	Web-based Cohort	105 healthy smokers	^a^ Urine biomarker.	Decreased ^c^: 66 to 98% of urine biomarkers of exposure (NNN, NNAL, 1-OHP, 3-HPMA, S-PMA, MHBMA, HMPMA).
Effects on body mass				
Veldheer et al. (34)	Randomized trial	263 smokers	^b^ Weight (pounds).	No significant differences at 1 month and 3 months in weight.
Polosa et al. (37)	Cohort	9 smokers – 12 never smokers	^b^ Body weight (kg).	No statistically significant changes in body weight and e-cigarette use were evident^d^.
Effects on fertility				
Harlow et al. (54)	Cohort	1,158 smokers – 3,428 never smokers	^b^ Fecundability (fecundity index).	^c^ Reduced non statistically significant on fecundity index.

^a^Acute effects of electronic cigarettes; ^b^Long-term effects of electronic cigarettes; ^c^nicotine attributed effects; ^d^Effects found with and without nicotine exposure. All effects described in the table as “increase” or “decrease” correspond to statistically significant results found in the included studies.

#### 3.4.2 Effects on body mass

Regarding effects on body mass and body weight, e-cigarette use apparently has no effect, both in the short term as found by Veldheer et al. in their randomized controlled study in which there were no statistically significant variations in weight (34), and in the long term as demonstrated by Polosa et al. in their prospective study with a follow-up of 3.5 years in which no statistically significant changes in body weight and e-cigarette consumption were evident, even in participants who consumed e-cigarettes with nicotine (37).

#### 3.4.3 Effects on fertility

Despite the vast panorama of studies in animal models, only one report was found on e-cigarette and fertility in humans. Harlow et al. (54) found a small effect from a cohort study in US women, determined by a fertility index = 0.84; 95% CI: 0.67, 1.06 in current versus never users of e-cigarettes.

## 4 Discussion

Since their emergence as an alternative to tobacco smoking cessation, electronic nicotine delivery systems (ENDS) have been gaining popularity globally, with increasing use among non-tobacco smoking groups such as youth and even children (7, 16, 55). In addition to their well-known yet controversial role in facilitating tobacco smoking cessation and the associated health benefits (8, 56–58), their growing commonality may foster erroneous beliefs about the harmlessness of e-cigarette use.

The industry has evolved rapidly, marked by indiscriminate use, which in less than two decades has led to the development of devices with various characteristics, including disposable options (59). These devices commonly use e-liquid, which contains various substances that may be flavored and may contain nicotine. Undoubtedly, these features have made these devices appealing to a broad audience and have complicated their study (60, 61).

Research on the effects of e-cigarette use faces challenges due to these variations. This review has attempted to systematize the findings from research that aims to objectively assess the effects of e-cigarette use on various organs and tissues, identifying 33 studies conducted in different settings and with various comparisons.

**Figure 2:**
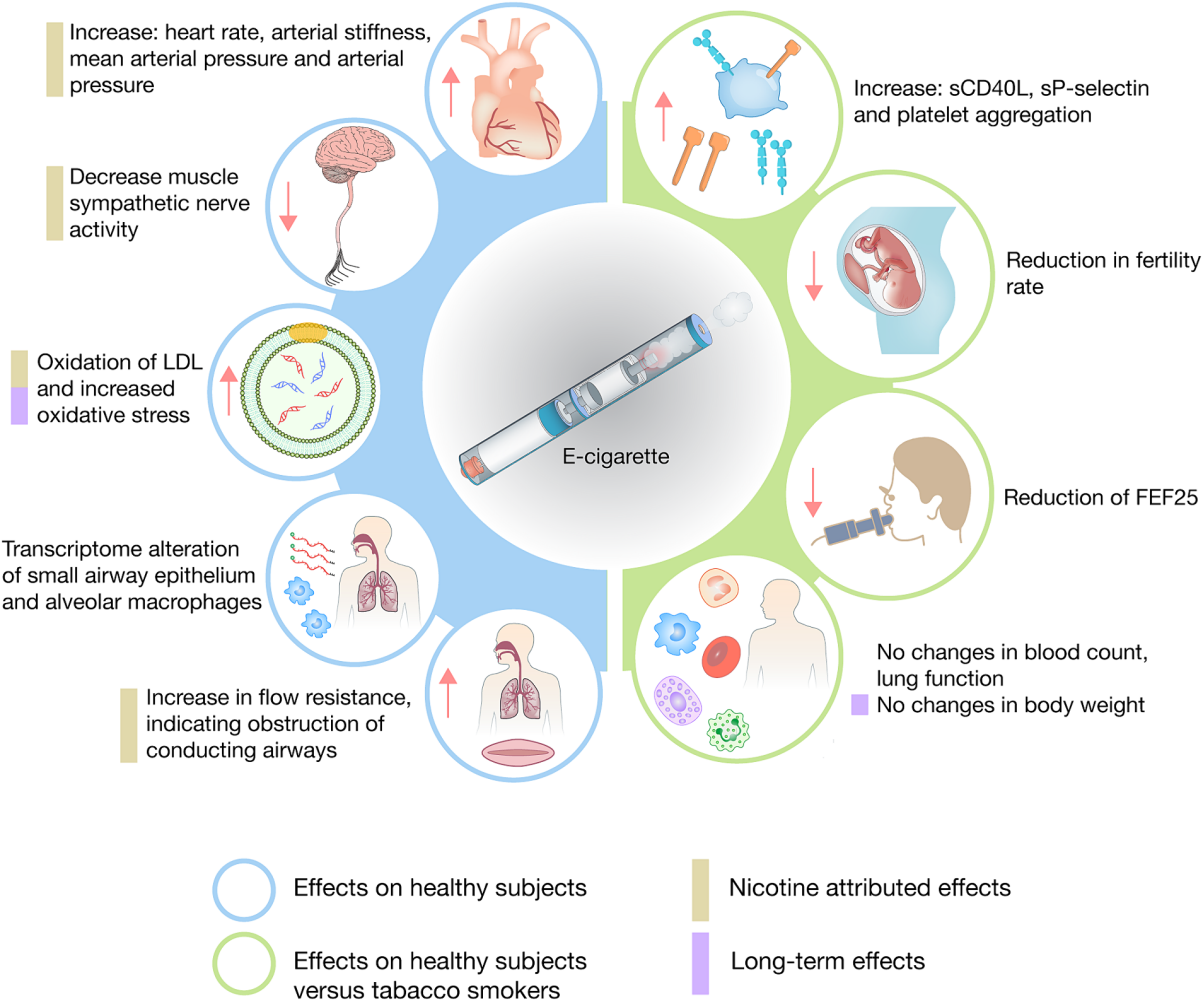
Synthesis of the investigated health effects of e-cigarettes in healthy individuals.

Although many experts support the use of electronic cigarettes, it is important to recognize that many positive findings on human health are based on comparisons with traditional cigarettes, as shown in this review. Of all the included studies, only seven evaluated the effects of electronic cigarettes in a healthy non-tobacco smoking population, these studies revealed significant effects, including increases in heart rate, mean arterial blood pressure, arterial stiffness, and oxidative stress, as well as respiratory system changes such as alterations in the transcriptomes of the respiratory epithelium and alveolar macrophages, and a sudden increase in airflow resistance suggesting airway obstruction. Many of these effects were attributed to the presence of nicotine compared to nicotine-free electronic cigarettes (22, 24, 27, 30, 45, 46, 48) (Figure 2). Additionally, five studies compared tobacco smokers with non-tobacco smokers, finding increases in vascular molecules and markers, including Scd40L, sP-selectin, platelet aggregation, significant reductions in pulmonary function (FEF-25), and a reduction in fertility incidence in women (37, 40, 47, 49, 54) (Figure 2).

The current literature included in this review discusses the acute effects of electronic cigarettes compared to traditional tobacco cigarettes. It is clear that most cigarette smokers find a risk reduction when switching to e-cigarettes. Although vaping is not harmless, it improves the health of those with chronic and continuous smoking. For instance, several studies highlighted positive outcomes associated with e-cigarette use; for instance, five studies reported reductions in blood pressure and heart rate among individuals who switched from smoking to using e-cigarettes, without immediate adverse effects on ventricular musculature or risk of acute myocardial infarction (32–34, 42, 43). However, three studies presented conflicting results on arterial stiffness (23, 33, 41). Increases in oxidative stress markers and endothelial and platelet vesicles were observed in terms of cardiovascular cellularity (23, 31, 39, 49). Respiratory health findings were predominantly positive, showing reductions in polycyclic aromatic hydrocarbons, specific tobacco nitrosamines, and improvements in symptoms such as cough, wheezing, and shortness of breath, with no significant changes in pulmonary function (32, 47, 51). Positive renal effects were noted in former smokers who switched to e-cigarettes, indicated by decreased urinary biomarkers of tobacco smoke exposure (52). Population differences, product use patterns, and study designs and methodologies contribute to the mixed results observed in research on the effects of electronic cigarettes (e-cigarettes) compared to traditional cigarettes (23, 33, 41). Some studies included healthy individuals, while others involved participants with pre-existing conditions. Additionally, the frequency and duration of e-cigarette use varied, impacting health outcomes. Variations in study designs, such as cross-over versus parallel designs, and differences in endpoints measured also influenced the results (23, 33, 41). Lastly, methodologies for measuring these endpoints, including techniques and timing of biomarker assessments, varied across studies. Despite these findings, it would be incorrect to universally deem the effects of e-cigarette use as positive. Future assessments should focus on healthy, non-smoking populations, as previous studies suggest that while e-cigarettes pose a lower cardiovascular risk than tobacco cigarettes, the risk remains significant (61, 62).

After carefully review and analyzed the literature, we found that some experts suggest that many of the harmful effects of e-cigarettes may be attributed to nicotine due to its direct impact and high addictiveness (63–65). Studies distinguishing between e-cigarettes with and without nicotine found increases in endothelial progenitor cells, endothelial vesicles, plasma myeloperoxidase, arterial stiffness, changes in nicotine receptors, alveolar macrophages, and mononuclear phagocytes, leading to altered inflammatory responses and increased pulmonary susceptibility in users of nicotine-containing e-cigarettes (29, 41, 46). These serious effects of nicotine are supported by the findings of Flouris et al., who noted the same cotinine levels in healthy non-tobacco smoking patients using nicotine e-cigarettes as in tobacco smokers (49). As this is a relatively new area of knowledge, the information is still conflicting. While some reviews emphasize the role of nicotine, other systematic reviews have shown that it is complicated to assign blame solely to nicotine (64, 66, 67).

Only five studies have sought to evaluate the long-term effects of e-cigarettes over periods ranging from 1 month to 3.5 years. These studies identified significant impacts on healthy participants with no smoking history, such as increased oxidability of low-density lipoproteins, decreased paraoxonase-1 activity, and lung inflammation attributed to propylene glycol (30, 45, 53). In contrast, among patients with a history of smoking, there were no significant changes in lung function 3 months after switching to e-cigarettes, although there was an increase in exhaled FeNO COe and improvements in clinical respiratory symptoms, including cough, phlegm, shortness of breath, and wheezing after 1 year (53). Comparisons between tobacco smokers and e-cigarette users showed no significant changes in body weight after 3.5 years of follow-up (37). The chronic effects of e-cigarettes remain unclear due to variability in study populations and a lack of consensus on what constitutes long-term exposure—ranging from as soon as 1 month to several years. Some experts believe the real long-term effects of e-cigarettes may only become apparent after decades (68).

Previous reviews have attempted to clarify the health effects of e-cigarettes, but this research addresses several identified limitations, including deficiencies in systematic search techniques and evidence evaluation. Notably, many studies did not differentiate between long and short-term effects or distinguish between populations that, despite being healthy, use tobacco (61, 68). The evaluation of evidence in this research exposed limitations, especially in the randomized controlled trials that assessed the effects of e-cigarettes. These trials showed deficiencies in treatment allocation (e-cigarette, tobacco, and placebo), comparative groups, and follow-up. Meanwhile, the cohort studies demonstrated relatively acceptable quality, with insufficient follow-up being their main limitation (Supplementary Tables 1–3).

The outlook for e-cigarettes on public health is concerning. This research underscores the need for further investigations to clarify misconceptions about e-cigarettes, including their real effects on populations not exposed to tobacco smoke, the impacts of long-term use, the effects of components other than nicotine like flavorings or moisturizers, and their actual effectiveness in smoking cessation (8).

The future concerns about e-cigarettes should mirror those historically associated with tobacco cigarettes. While this review focuses on the direct health impacts of e-cigarettes, it is crucial not to overlook their potential to attract very young non-smokers to substance use, thereby affecting public health significantly. We now can mitigate these long-term effects on global health and particularly on younger populations.

## 5 Conclusion

While traditional cigarettes are harmful to health, vaping offers some risk reduction. However, notable adverse effects, especially from nicotine-containing e-cigarettes on cardiovascular and respiratory function, challenge the perception that e-cigarettes are harmless. Studies in healthy non-smokers show significant adverse outcomes, suggesting e-cigarettes are not safe for non-smokers and could be harmful long-term. Moreover, the long-term effects remain uncertain, with potential risks similar to traditional smoking.

This review highlights key findings: the impact of e-cigarettes on oxidative stress, endothelial function, and platelet activation varies with smoking history and health status. Synthesizing data across smokers, ex-smokers, and non-smokers provides a comprehensive overview not covered in previous reviews. By considering these differences, our review offers a nuanced understanding of e-cigarette impacts. The analysis of markers and outcomes provides new insights into potential health risks, emphasizing the need for targeted public health policies. This information is crucial, especially for non-smokers and youth misled by e-cigarette safety profiles and flavors. Our review underscores the need for more research to define e-cigarette health impacts, including long-term effects and the impact of various e-liquid components.

## Data availability statement

The original contributions presented in the study are included in the article/Supplementary material, further inquiries can be directed to the corresponding author.

## Author contributions

JI-C: Methodology, Project administration, Resources, Software, Supervision, Validation, Visualization, Writing – original draft, Writing – review & editing, Conceptualization, Data curation, Formal analysis, Investigation. PN-L: Data curation, Formal analysis, Investigation, Methodology, Resources, Software, Visualization, Writing – original draft. EM-L: Conceptualization, Data curation, Formal analysis, Investigation, Methodology, Resources, Validation, Visualization, Writing – original draft. MH: Data curation, Formal analysis, Investigation, Methodology, Resources, Software, Visualization, Writing – original draft. AT-D-l-T: Data curation, Formal analysis, Investigation, Methodology, Resources, Visualization, Writing – original draft. EV-G: Data curation, Investigation, Visualization, Writing – original draft. CS-S: Formal analysis, Investigation, Methodology, Project administration, Software, Visualization, Writing – original draft. VL-G: Data curation, Investigation, Project administration, Validation, Writing – original draft. WR: Data curation, Formal analysis, Investigation, Resources, Writing – original draft. DB: Data curation, Formal analysis, Investigation, Resources, Validation, Writing – original draft. MG: Data curation, Formal analysis, Investigation, Resources, Validation, Writing – original draft. AL-C: Investigation, Resources, Supervision, Validation, Visualization, Writing – original draft, Writing – review & editing. EO-P: Investigation, Methodology, Project administration, Resources, Validation, Visualization, Writing – review & editing.
