# Supplementary material for: Direct health implications of e-cigarette use: a systematic scoping review with evidence assessment
Source: Front Public Health. 2024 Jul 29;12:1427752. doi: 10.3389/fpubh.2024.1427752 (PMC11317248; doi:10.3389/fpubh.2024.1427752)
Supplement: Supplementary file 1 [file Table_1.docx]

**Table S1:** Newcastle - Ottawa quality assessment scale for *Cohort Studies*

| **Study** | **1** | **2** | **3** | **4** | **5** | **6** | **7** | **8** | **Score** |
| --- | --- | --- | --- | --- | --- | --- | --- | --- | --- |
| Antoniewicz L. et al., 2019 (13) | * | * | * | * | ** | * | * | * | 9 |
| Farsalinos K. et al., 2016 (24) | * | * | * | * | ** | * | * | * | 9 |
| George J. et al., 2019 (22) | * | * | * | * | ** | * | * | * | 9 |
| Gonzalez JE. et al., 2021 (11) | * |  | * | * | * | * | * | * | 7 |
| Spindle TR. et al., 2018 (17) | * |  | * | * | * | * |  | * | 6 |
| Mobarrez F. et al., 2020 (27) | * | * | * | * | ** | * | * | * | 9 |
| Nocella C. et al., 2018 (28) | * | * | * | * | ** | * | * | * | 9 |
| Campagna D. et al., 2016 (37) | * | * | * | * | ** | * | * | * | 8 |
| Cioe P. et al., 2020 (39) | * |  | * | * | * | * | * | * | 7 |
| D’Ruiz C. et al., 2017 (21) | * | * | * | * | * | * | * | * | 8 |
| Dicpinigaitis P. et al., 2016 (38) | * | * | * | * | * | * | * | * | 8 |
| Kerr D. et al., 2019 (20) | * | * | * | * | * | * | * | * | 8 |
| Staudt M. et al., 2018 (33) | * | * | * | * | * | * |  |  | 6 |
| Harlow A. et al., 2020 (42) | * | * | * | * | ** | * | * | * | 9 |
| Polosa R. et al., 2017 (41) | * | * | * | * | ** | * | * | * | 9 |

**Note:** A study can receive a maximum of one star for each item numbered within the Selection and Result categories. A maximum of two stars can be awarded for comparability.

**Selection**

1. Representativeness of the exposed court.

2. Selection of the unexposed court.

3. Exposure determination.

4. Demonstration that the current outcome of interest was not present at baseline.

**Comparability**

5. Cohort comparability based on design or analysis.

**Results**

6. Evaluation of the result.

7. Was the follow-up long enough for the results to occur?

8. Adequacy of cohort follow-up.

**Interpretation**

Good quality: 3 or 4 stars in the selection domain and 1 or 2 stars in the comparability domain and 2 or 3 stars in the outcome/exposure domain.

Acceptable quality: 2 stars in the selection domain and 1 or 2 stars in the comparability domain and 2 or 3 stars in the outcome/exposure domain.

Poor quality: 0 or 1 star in the selection domain or 0 stars in the comparability

domain or 0 or 1 stars in the outcome/ exposure domain

**Table S2:** JBI critical appraisal checklist for Randomized Controlled Trials

| **Study** | **1** | **2** | **3** | **4** | **5** | **6** | **7** | **8** | **9** | **10** | **11** | **12** | **13** | **Score** |
| --- | --- | --- | --- | --- | --- | --- | --- | --- | --- | --- | --- | --- | --- | --- |
| Biondi-Zoccai G. et al., 2019 (12) | YES | YES | YES | YES | YES | YES | YES | YES | YES | YES | YES | YES | YES | 13 |
| Yan XS. Et al., 2015 (14) | YES | NO | NO | YES | YES | NO | NO | NO | NO | YES | NO | YES | YES | 6 |
| Dimitriadis K. et al., 2022 (15) | YES | YES | YES | YES | YES | NO | NO | YES | YES | YES | YES | YES | YES | 11 |
| Chaumont M. et al., 2018 (18) | YES | YES | YES | YES | YES | YES | YES | YES | YES | YES | YES | YES | YES | 13 |
| Moheimani RS. et al., 2017 (19) | YES | NO | YES | NO | NO | NO | YES | YES | YES | YES | YES | YES | YES | 9 |
| D’Ruiz CD. et al., 2017 (21) | YES | YES | YES | NO | NO | NO | YES | YES | YES | YES | YES | YES | YES | 10 |
| Klonizakis M. et al., 2022 (25) | YES | YES | YES | YES | YES | NO | NO | NO | YES | YES | YES | YES | YES | 12 |
| Song M. et al., 2020 (32) | YES | YES | YES | NO | NO | YES | YES | YES | YES | YES | YES | YES | YES | 11 |
| Veldheer S. et al., 2019 (23) | YES | YES | YES | YES | NO | YES | YES | YES | YES | YES | YES | YES | YES | 12 |
| Chaumont M. et al., 2020 (35) | YES | YES | YES | YES | YES | YES | YES | YES | YES | YES | YES | YES | YES | 13 |
| Antoniewicz L. et al., 2016 (29) | YES | NO | NO | NO | NO | NO | NO | YES | NO | YES | YES | YES | YES | 6 |
| Flouris AD. Et al., 2012 (26) | YES | NO | NO | NO | NO | NO | YES | NO | YES | YES | YES | YES | YES | 7 |
| Farsalinos K. et al., 2014 (30) | NO | NO | NO | NO | NO | YES | YES | YES | NO | YES | YES | YES | YES | 7 |
| Ferrari M. et al., 2015 (34) | YES | NO | NO | NO | NO | NO | NO | YES | YES | YES | YES | YES | YES | 7 |
| Flouris A. et al., 2013 (36) | NO | NO | NO | NO | NO | NO | YES | YES | NO | YES | YES | YES | NO | 5 |
| O’Connell G. et al., 2016 (40) | YES | NO | NO | NO | NO | NO | YES | YES | YES | YES | YES | YES | NO | 7 |
| Vansickel A. et al., 2010 (43) | YES | YES | YES | NO | NO | NO | YES | YES | YES | YES | YES | YES | YES | 10 |

1. Was true randomization used for assignment of participants to treatment groups?

2. Was allocation to treatment groups concealed?

3. Were treatment groups similar at the baseline?

4. Were participants blind to treatment assignment?

5. Were those delivering treatment blind to treatment assignment?

6. Were outcomes assessors blind to treatment assignment?

7. Were treatment groups treated identically other than the intervention of interest?

8. Was follow up complete and if not, were differences between groups in terms of their follow up adequately described and analyzed?

9. Were participants analyzed in the groups to which they were randomized?

10. Were outcomes measured in the same way for treatment groups?

11. Were outcomes measured in a reliable way?

12. Was appropriate statistical analysis used?

13. Was the trial design appropriate, and any deviations from the standard RCT design (individual randomization, parallel groups) accounted for in the conduct and analysis of the trial?

**Interpretation**

Quality scores were categorized into three groups: Low: 1-4, Moderate: 5-7, and High: >8

**Table S3:** JBI critical appraisal checklist for analytical cross-sectional studies

| **Study** | **1** | **2** | **3** | **4** | **5** | **6** | **7** | **8** | **Score** |
| --- | --- | --- | --- | --- | --- | --- | --- | --- | --- |
| Alzahrani T. et al., 2018 (31) | YES | YES | YES | YES | YES | YES | YES | YES | 8 |

1. Were the criteria for inclusion in the sample clearly defined?

2. Were the study subjects and setting described in detail?

3. Was the exposure measured in a valid and reliable way?

4. Were standard and objective criteria used to measure the condition?

5.Were confounding factors identified?

6. Were strategies established to deal with confounding factors?

7. Were the outcomes measured in a valid and reliable way?

8. Was an appropriate statistical analysis used?

**Interpretation**

Quality scores were categorized into three groups: Low: 1-4, Moderate: 5-7, and High: >8
